# Forearm bone mineral density in adult men after spinal cord injuries: impact of physical activity level, smoking status, body composition, and muscle strength

**DOI:** 10.1186/s12891-022-05022-4

**Published:** 2022-01-24

**Authors:** Anna Kopiczko, Joanna Cieplińska

**Affiliations:** 1 Department of Human Biology, Józef Piłsudski University of Physical Education in Warsaw, Marymoncka St. 34, 00-968 Warsaw, Poland; 2 Department of Physiotherapy, College of Rehabilitation, Kasprzaka St. 49, 01-234 Warsaw, Poland

## Abstract

**Background:**

In the present cross-sectional study, we analyzed the relationships of physical activity level, muscle strength, body composition, injury parameters, and smoking status with bone health in the non-paralyzed upper limb in adult men after spinal cord injuries (SCI).

**Methods:**

The study covered 50 men after spinal cord injuries aged 35.6 ± 4.9 years (25 wheelchair rugby players and 25 non-athletes). Forearm bone mineral density (BMD), bone mineral content (BMC) in distal (dis) and proximal (prox) part was measured by densitometry. Body mass index (BMI) and body fat percentage (BF) were calculated. Fat mass (FM) and fat-free mass (FFM) were estimated from somatic data. An interview was conducted based on the Global Adult Tobacco Survey questionnaire. Muscle strength (maximal hand grip strength) was measured using a Jamar dynamometer.

**Results:**

Active male smokers after SCI had significantly lower BMD dis, BMC dis and prox, T-score dis, and prox (large effect > 0.8) than male non-smokers after SCI. Physical activity was a significant predictor (positive direction) for BMC prox (adjusted R2 = 0.56; *p* < 0.001). The predictor of interactions of physical activity and fat mass was significant for BMC dis (positive direction, adjusted R2 = 0.58; p < 0.001). It was also found that the predictor of interactions of four variables: physical activity, fat mass, hand grip strength (positive direction), and years of active smoking (negative direction) was significant for BMD dis (adjusted R2 = 0.58; *p* < 0.001). The predictor of interactions of age at injury (additive direction) and the number of cigarettes smoked per day (negative direction) was significant for T-score prox (adjusted R2 = 0.43; p < 0.001). Non-smoking physically active men after SCI had the most advantageous values of mean forearm BMD.

**Conclusion:**

Rugby can be considered a sport that has a beneficial effect on forearm BMD. The physically active men after SCI had significantly higher bone parameters. Physical activity itself and in interactions with fat mass, hand grip strength (positive direction), and years of active smoking (negative direction) had a significant effect on bone health in non-paralyzed upper limbs. Active smoking may reduce the protective role of physical activity for bone health.

## Background

Spinal cord injury (SCI) is a disabling neurological condition resulting in severe dysfunctions of the entire body [1]. Osteoporosis is a debilitating secondary complication of complete SCI [2, 3], whereas insufficient BMD mainly affects the paralyzed limbs. Studies suggest that the demineralization process in these patients proceeds rapidly for the first few months, reaching a peak around weeks 10 to 16. Then, the process stabilizes until it reaches equilibrium between 12 and 36 months after the injury. Recent studies, however, indicate that the process does not stabilize until approximately 7 to 8 years after injury [1–4].

Bone mineral density (BMD) in the different parts of the skeleton and limbs declines precipitously in the first 2 years after SCI. BMD varies by region [1–7], for example the loss of BMD in the femur and tibia during the first year after SCI can occur significantly earlier than that of the hip BMD [1, 5]. The rate of bone mass loss shows large interindividual differences. There are few data on BMD changes in the upper limb including the forearm [8]. For SCI patients, it is the upper limbs that become the primary driver of wheelchair locomotion. It is recommended to monitor BMD in early-stage SCI patients combined with the identification of factors leading to lower BMD [1].

Physical activity (PA) after a spinal cord injury is considered to be an important factor in rehabilitation or a means to enable maximum independence, but its importance in preventing deterioration of bone tissue is also indicated [9, 10]. During physical exercise, bone adapts to mechanical loads generated by e.g. muscle contraction. A direct effect of muscle mass on bone structure and bone strength is known [11]. Muscle strength and hand grip strength (HGS) may be associated with the increasing BMD in men after SCI [12]. In a healthy male population, HGS has been shown to be associated with forearm BMD [13, 14]. The effect of the physical activity of wheelchair athletes on BMD is of growing interest to scientists. However, the findings vary. This is because various factors affecting BMD of wheelchair players with SCI have been analyzed, including age, somatic features (body weight, tissue composition) [15, 16], type of sport [10, 17], area of injury, period of injury, and time needed to resume sporting activity after the injury.

Importantly, it has been demonstrated that the sooner patients with SCI started sports training after rehabilitation, the faster the higher BMD values were recorded in the lower limbs, trunk, and skeleton regardless of age and sport [10]. However, there is a lack of research on the effects of active lifestyles on the condition of the bones of the non- paralyzed limbs and other factors that often have an opposite effect on the skeletal system. For example, in the case of athletes, active smoking (AS) is rarely taken into account in health analyses, and studies have shown that this population uses such stimulants. Nicotine use among athletes is high and increasing, especially in team sports [18]. Few studies have evaluated the effect of AS on bone tissue in athletes. In general, cigarette smoking has been identified as a factor in the reduction of bone mineral content (BMC), consequently increasing the risk of fractures in the general population. The relationship between AS (and also passive smoking (PS) in the form of exposure to environmental smoke) and bone health is explained by the deterioration of bone strength caused by harmful substances contained in tobacco. It is also indicated that this phenomenon is more pronounced in men than in women [19–21]. A meta-analysis referring to the effect of smoking on bone condition suggested a reduction of bone mass in active smokers compared to non-smokers. Studies also show a greater decline in BMC among smoking men compared to smoking women [22]. Scientists have discovered that the smoker’s body synthesizes extremely large amounts of two proteins S100A8 and S100A9, which are conducive to osteoclast production. Researchers believe that this is what lies behind the negative effect of AS on bone density [23].

It is still unclear whether factors such as physical activity, high strength, and good body composition can have a protective effect on bone tissue and reduce the risk of osteopenia and osteoporosis in later years of life after SCI. Given the multifactorial determinants of BMD and only few studies on the effect of smoking on bone tissue in athletes after SCI, in this cross-sectional study, we analyzed the relationships of physical activity level, muscle strength, body composition, injury parameters, and smoking status with bone health in the non-paralyzed upper limbs, in adult men after SCI.

## Methods

### Participants and data collection

The present cross-sectional study covered 50 men aged 35.6 ± 4.9 years. The first group consisted of 25 men with SCI, who were players from a wheelchair rugby team. Based on the ASIA impairment scale (AIS) there were 10 men in Grade A (Type of Injury: complete) and 15 men in Grade B (Type of Injury: Incomplete sensors) in this group [24]. Training experience was from 5 to 11 years of organized wheelchair rugby training. The frequency of training in this group was 4 training sessions per week including one body weight circuit strength session using sports equipment. Before SCI, the subjects had not practiced any sport. The second group who volunteered to participate in the study consisted of 25 men of similar age with spinal cord injuries, moving in wheelchairs, and physically inactive (non-athletes). Based on the AIS classification, this group included 13 men in Grade A (Type of Injury: complete) and 12 men in Grade B (Type of Injury: Incomplete sensors). Information was obtained from clinical history and interviews with the men about age at injury and the period of injury [25]. The inclusion criterion was the lack of contraindications to densitometric examination, written informed consent to participate in the study, the absence of diseases affecting bone metabolism such as thyroid diseases, rheumatoid arthritis, chronic steroid treatment, or rickets. All of the participants were informed about the aims, benefits, and procedures of the research project, and the possibility to withdraw from the experiment at any moment without providing an explanation. The inclusion criterion was also the written consent of the participant.

### Measures

#### Kinanthropometric measurements

Somatic measurements were performed in accordance with the kinanthropometric methodology adopted in measurements of disabled people [26]. Height was measured with an anthropometer (GPM Anthropometer Siber Hegner, Switzerland) with a precision of 0.1 cm in a supine position. Body weight of the participants was obtained with a wheelchair medical scale. First, the men were measured in a wheelchair, and then the wheelchair weight was measured separately. Body weight of each athlete was calculated as the difference between these measurements. Body mass index (BMI) was also calculated. The BMI classification was adopted in accordance with the recommendation of the World Health Organization (WHO). The percentage of body fat (BF%) was calculated according to Deurenberg et al. [27] using the formula for men. Fat mass (FM in kg) and fat-free mass (FFM in kg) were estimated from somatic measurements using formulas adjusted to age, gender, and the European white population [28].

### Methods of bone tissue evaluation

BMC and BMD of the non-dominant forearm in distal (dis) and proximal (prox) parts were measured by means of dual-energy X-ray absorptiometry (DXA, Norland, Swissray, Fort Atkinson, WI, USA) The Norland DXA instruction recommends two measurement points: on the ^1^/_3_ proximal (prox) (radius + ulna) and distal (radius + ulna) sites of the bone according to the adopted densitometry methodology and the recommendations of the International Society for Clinical Densitometry (ISCD). The test protocol was Bone Mineral Density Testing in Spinal Cord Injury [3]. The DXA data were used to calculate the T-score. The WHO definition of osteoporosis is based on the T-score. The T-score is a comparison of a patient’s BMD to that of a healthy thirty-year-old person of the same sex and ethnicity (expressed in standard deviations). T-scores of − 1 and above were considered normal, while T-scores between − 1 and − 2.5 were considered low BMD [29]. The study was conducted in all people using the same equipment by a team having the necessary qualifications and experience in the research using the above-mentioned method and apparatus. The scanner was calibrated daily against the standard calibration block supplied by the manufacturer (QA Phantom type S/N, QC Phantom MD: 0.825) to control for possible baseline drift. Low patient dose of less than 1.8 mRems per scan high speed. Low scatter radiation of < 0.1 mRem per hour 1.0 m from beam. The scanning area was 150 mm × 125 mm. The resolution was 1 mm × 1 mm. X-ray Source is tin filtered, 60 kV and X-ray Energies is 28 keV and 48 keV [30].

### Measurements of muscle strength

Maximal hand grip strength (HGS) was measured in the non-dominant hand using a Jamar dynamometer and following a standardized protocol [31]. The measurements were repeated twice, with brief pauses between them the best result was considered the maximum HGS.

### Assessment of smoking

An interview method using the Global Adult Tobacco Survey (GATS) Questionnaire Section B (Tobacco Smoking) and Section D1 (Cessation: Tobacco Smoking) was employed. This survey assessed quantitative variables such as the total number of years of active smoking (AS) and the number of cigarettes smoked per day. A standard global protocol was used to implement GATS. The examination methodology was consistent with the guidelines of experts of the World Health Organization [32].

### Statistical analysis

All the calculations and analyses were performed using the STATISTICA software (v.12, Stat. Soft., the USA). Student’s t-test for independent variables was applied to determine the significance of differences between the values of particular variables for active and inactive men with SCI and male smokers and non- smokers with SCI. Effect size was calculated using Cohen’s d = 2 t / (df^1/2), (small effect: < 0.5; medium effect: 0.5–0.8; large effect: > 0.8). Differences between the frequency of low and normal BMD were analyzed using the chi-squared test. Two-way ANOVA analysis was applied to determine the relationships of PA category and AS with BMD. A measure of the effect size eta square (η2) is given. The multiple forward stepwise regression model was applied to determine the relationships between bone parameters (BMD, BMC, and T-score) in the distal and proximal segments and individual predictor variables. Statistical significance was set at *p ≤ 0.05, **p ≤ 0.01 and ***p ≤ 0.001.

## Results

The basic characteristics of the two groups of men with SCI (active and inactive; active smokers and non-smokers) in terms of biometric and somatic characteristics, smoking status, maximal hand grip strength, the significance of differences, and effect size calculated using Cohen’s d are presented in Table 1. The groups differed significantly in 7 of 12 analyzed parameters. The active men after SCI were slightly older, had been older when the injury occurred, taller, had lower BMI (medium effects: 0.5–0.8), smaller BF (small effect < 0.5), smaller FM (large effect > 0.8), and higher maximal hand grip strength (large effect > 0.8).

**Table 1:** Characteristics of study population (*n* = 50)

	**Active men after SCI- Wheelchair rugby players (*n* = 25)**	**Inactive men after SCI (*n* = 25)**	***P*-value**	**Cohen’s *d***
	**mean ± SD**			
Age (years)	36.6 ± 5.1	33.9 ± 3.5	0.037*	0.617
Age when injury occurred (years)	23.0 ± 5.1	20.3 ± 2.6	0.019*	0.667
The period of injury (years)	13.5 ± 7.3	13.6 ± 2.9	0.949	0.018
Weight (kg)	77.5 ± 14.0	79.7 ± 11.6	0.548	0.171
Height (cm)	182.2 ± 7.7	176.9 ± 6.8	0.014**	0.729
BMI (kg/m^2^)	23.3 ± 3.4	25.4 ± 3.1	0.026*	0.645
BF (%)	21.1 ± 4.6	22.5 ± 3.7	0.049*	0.311
FM (kg)	16.1 ± 6.6	23.0 ± 3.0	0.000***	1.345
FFM (kg)	61.4 ± 8.3	57.7 ± 10.6	0.182	0.388
HGS (kg)	104.0 ± 11.2	92.8 ± 11.5	0.002**	0.944
AS (years)	2.6 ± 3.7	4.2 ± 5.1	0.226	0.359
AS (cigarette/day)	4.1 ± 5.3	3.9 ± 4.8	0.225	0.039
	**Active smokers men after SCI (*n* = 21)**	**Non-smokers men after SCI (*n* = 29)**	***P*-value**	**Cohen’s** ***d***
	**mean ± SD**			
Age (years)	35.0 ± 4.4	35.4 ± 4.6	0.705	0.089
Age when injury occurred (years)	20.5 ± 5.1	22.5 ± 3.3	0.094	0.466
The period of injury (years)	15.1 ± 6.3	12.5 ± 4.6	0.101	0.471
Weight (kg)	74.0 ± 10.5	82.8 ± 12.4	0.012**	6.563
Height (cm)	176.4 ± 8.1	181.9 ± 6.6	0.011**	0.744
BMI (kg/m^2^)	23.8 ± 2.7	25.1 ± 3.8	0.202	0.394
BF (%)	20.5 ± 3.6	21.9 ± 4.8	0.276	0.329
FM (kg)	19.5 ± 5.8	19.7 ± 6.1	0.901	0.034
FFM (kg)	54.6 ± 9.1	63.1 ± 8.3	0.001***	0.976
HGS (kg)	95.5 ± 10.1	100.5 ± 14.6	0.180	0.398

*AS* active smoking, *BMI* Body Mass Index, *BF* Body Fat, *FM* Fat Mass, *FFM* Fat Free Mass, *HGS* Hand Grip Strength; Student’s t-test *P* value reported for continuous variables. Statistical significance was set at the levels of *p ≤ 0.05, **p ≤ 0.01 and ***p ≤ 0.001

The active smokers men after SCI were statistically significant lower body weight (large effect > 0.8) and height (medium effects: 0.5–0.8), and FFM (large effect > 0.8) compared to non-smokers men after SCI (Table 1).

Characteristics of bone parameters in men with SCI, the level of physical activity, and smoking status are presented in Table 2. The active men with SCI had significantly higher bone parameters such as BMD prox, BMC dis and prox, and T-score prox (large effect d > 0.8). The male smokers differed significantly in 5 of 6 analyzed forearm bone parameters. Male active smokers with SCI had significantly smaller BMD dis, BMC dis and prox, T-score dis, and prox (large effect > 0.8) than male non-smokers after SCI (Table 2).

**Table 2:** Characteristics of men’s bone parameters including physical activity and smoking status

	**Active men after SCI Wheelchair rugby players (*n* = 25)**	**Inactive men after SCI (*n* = 25)**	***P*-value**	**Cohen’s *d***
	**mean ± SD**			
BMD dis (g/cm^2^)	0.454 ± 0.095	0.412 ± 0.057	0.064	0.536
BMD prox (g/cm^2^)	0.905 ± 0.089	0.792 ± 0.178	0.007**	0.803
BMC dis (g)	2.185 ± 0.380	1.696 ± 0.176	0.000***	1.651
BMC prox (g)	2.710 ± 0.381	2.069 ± 0.429	0.000***	1.579
T-score dis	0.051 ± 1.419	−0.560 ± 1.578	0.156	0.407
T-score prox	−0.996 ± 1.003	−1.795 ± 0.992	0.007**	0.801
	**Active smokers men after SCI (*n* = 21)**	**Non-smokers men after SCI (*n* = 29)**	***P*-value**	**Cohen’s** ***d***
	**mean ± SD**			
BMD dis (g/cm^2^)	0.393 ± 0.051	0.462 ± 0.086	0.002**	0.976
BMD prox (g/cm^2^)	0.816 ± 0.081	0.872 ± 0.183	0.193	0.396
BMC dis (g)	1.764 ± 0.246	2.069 ± 0.417	0.004**	0.891
BMC prox (g)	2.128 ± 0.400	2.578 ± 0.513	0.002**	0.978
T-score dis	−1.395 ± 0.644	0.571 ± 1.432	0.000***	1.771
T-score prox	−2.013 ± 0.572	−0.948 ± 1.124	0.000***	1.194

*BMD* Bone Mineral Density, *BMC* Bone Mineral Content, prox- proximal part of forearm, dis- distal part of forearm; Student’s t-test *P* value reported for continuous variables. Statistical significance was set at the levels of *p ≤ 0.05, **p ≤ 0.01 and ***p ≤ 0.001

There were significant differences (*p* < 0.01) in the prevalence of low bone mineralization of the forearm depending on physical activity level and smoking status in men with SCI. A significantly higher (nearly double) percentage of individuals with low BMD was found for inactive compared to active men. Significant differences (*p* < 0.001) in the prevalence of low BMD were observed also between smokers and non-smokers (Table 3).

**Table 3:** The frequency of low bone mineral status in active and inactive men after SCI, smokers and non-smokers men after SCI (*Chi2 test, level of significance p*)

	**Reference ranges**	**Active men after SCI Wheelchair rugby players**	**Inactive men after SCI**	**Chi** ^**2**^ **(*p*)**
		**%**		
BMD dis	**Low BMD**	**20**	**56**	6.876 (0.009)**
	Norm BMD	80	44	
BMD prox	**Low BMD**	**44**	**80**	7.089 (0.007)**
	Norm BMD	56	20	
		**Active smokers men after SCI**	**Non-smokers men after SCI**	
		**%**		
BMD dis	**Low BMD**	**71.4**	**13.8**	17.173 (0.000)***
	Norm BMD	28.6	86.2	
BMD prox	**Low BMD**	**100**	**34.5**	22.191 (0.000)***
	Norm BMD	0	65.5	

*BMD* Bone Mineral Density, prox- proximal part of forearm, dis- distal part of forearm. Low BMD - T-score < − 1; Statistical significance was set at the levels of *p ≤ 0.05, **p ≤ 0.01 and ***p ≤ 0.001

The results of the multiple forward stepwise regression analysis are presented in Table 4. The presented model explained 23–58% (adjusted R2 = 0.23-0.58; *p* < 0.001) of the variance in bone parameters. Physical activity itself was a significant predictor (positive value of the standardized β coefficient) for BMC prox (adjusted R2 = 0.56; p < 0.001). The predictor of interactions of physical activity and fat mass was significant for BMC dis (positive direction, adjusted R2 = 0.58; *p* < 0.001). It was also found that the predictor of interactions of four variables: physical activity, fat mass and hand grip strength (positive direction) as well as years of active smoking (negative direction) was significant for BMD dis (adjusted R2 = 0.58; *p* < 0.001). Furthermore, the predictor of interactions age when injury occurred (additive direction) and number of cigarettes smoked per day (negative direction) was significant for T-score prox (adjusted R2 = 0.43; p < 0.001), (Table 4).

**Table 4:** Relationships between bone mineral density (BMD), bone mass (BMC) and T-score in the distal and proximal part of the forearm and somatic features, body compositions, smoking parameters, maximal hand grip strength, the level of physical activity and injury parameters – multiple forward stepwise regression

Bone parameters	Predictor	Standardized β	Adjusted R^2^	F (p)
**BMD dis**	BMI	−0.338	0.39	7.15 (< 0.000)
	FM	0.767		
	HGS	0.339		
	PA	0.371		
	ASy	−0.420		
**BMD prox**	FFM	0.231	0.23	3.863 (< 0.005)
	HGS	0.213		
	PA	0.194		
	AI	0.137		
	PI	−0.168		
**BMC dis**	FM	0.367	0.58	14.772 (< 0.000)
	FFM	−0.159		
	PA	0.862		
	ASy	−0.194		
	ASc/d	−0.258		
**BMC prox**	FM	0.224	0.56	16.319 (< 0.000)
	PA	0.727		
	ASy	−0.208		
	ASc/d	−0.209		
**T-score dis**	Age	0.164	0.48	10.075 (< 0.000)
	FFM	0.221		
	HGS	0.161		
	ASy	−0.236		
	ASc/d	−0.313		
**T-score prox**	HGS	0.125	0.43	10.113 (< 0.000)
	PA	0.227		
	AI	0.312		
	ASc/d	−0.406		

*BMI* body mass index, *FM* fat mass, *FFM* fat-free mass, *HGS* hand grip strength, *PA* physical activity, *ASy* years of active smoking, *ASc/d* active smoking cigarette/day, *AI* Age when injury occurred, *PI* The period of injury

**Fig. 1:**
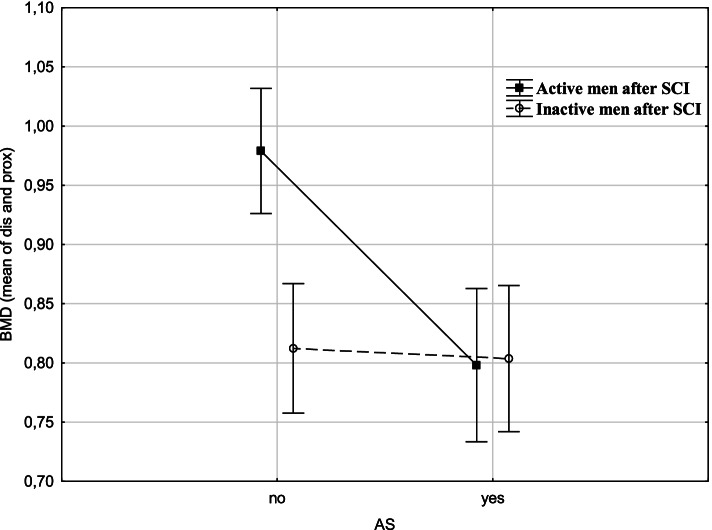
Relationships of PA category and AS with BMD dis (results two-way ANOVA analysis; F = 87.140; p=.0049; η^2^ = 0.16), vertical lines − 0.95 CI - confidence intervals

Fig. 1 presents a graphical representation of the results of the analysis of variance. Non-smoking active men with SCI had the most advantageous values of mean BMD in the forearm. Smoking, especially among physically active men with SCI, corresponded to significantly lower BMD (by 0.20 g/cm2). Among inactive men with SCI, the effect of smoking on mean BMD was less pronounced. The interaction of variables physical activity and smoking explains 16% (η2 = 0.16) of the variance (Fig. 1).

## Discussion

The most important finding of this study to be that smoking appears to undermine the otherwise salutary effect of physical activity on forearm BMD. Smoking, especially among physically active men with SCI, corresponded to significantly lower BMD. In our study, in addition to smoking we analyzed concerned the impact of physical activity (wheelchair rugby training), body tissue components (fat mass and fat-free mass), age at injury, and the period of injury on forearm bone parameters. In our study, the prevalence of low BMD in wheelchair rugby players was lower than in inactive men. In both groups of men with SCI, low BMD was particularly pronounced in the proximal part of the forearm.

In the present study, physical activity was a significant positive predictor for BMC prox. The predictor of interactions of physical activity and fat mass was also significantly positive for BMC dis. It was found that the predictor of interactions of four variables: physical activity, fat mass, hand grip strength (positive direction), and years of active smoking (negative direction) was significant for BMD dis. Furthermore, the predictor of interactions of age at injury (additive direction) and the number of cigarettes smoked per day (negative direction) was significant for T-score prox.

The condition of forearm bone tissue in men with SCI has not been studied previously [8]. The research to date has focused on the evaluation of BMD in patients with SCI depending on their lifestyles, physical activity levels [10, 33], diets, and supplementation [34]. Studies have evaluated body composition in relation to BMD and BMC in men with SCI [15, 35]. The determinants of fracture risk have often been analyzed in individuals with spinal cord injury [36]. Research has shown that bone loss in individuals with SCI occurs especially in the lower limbs. No significant changes in BMD caused by SCI were found in the proximal and distal parts of the radial bone of the forearm [8, 33, 37]. However, active men with SCI who often performed movements engaging upper limbs (e.g. wheelchair basketball players) had higher BMD than inactive men with SCI [9]. Goktepe et al. [9] compared bone mineral density in elite paraplegic basketball players with values obtained for paraplegic sedentary controls. Wheelchair basketball players with spinal cord injuries were characterized by greater bone density in the distal radius compared to sedentary paraplegic patients. Our research of wheelchair rugby players also showed higher BMD compared to non-active men with SCI. A higher percentage of low BMD cases (by 36%) was found for inactive men at both bone points. Eloumi et al. [38] examined the effects of long-term rugby participation on bone mineral content (BMC) and density (BMD) in male rugby players and attempted to determine whether different stimuli elicited by actions of forwards and backs affect their skeleton differently. These researchers showed that long-term rugby participation, starting at pubertal age, is associated with markedly increased BMC, BMD, and bone size at all skeletal sites, except at the head. Similarly, in our study, men performed rugby training 5 years or longer, and, as analyses show, had significantly better BMD than inactive men. A previous BMD study of the forearm of Polish healthy young men showed lack of PA is clearly associated with an increased occurrence of osteopenia and osteoporosis in men. The men with sufficient to high levels of PA demonstrated normal values for the T-score in the both the distal and proximal forearm measurement sites in more than 90% of participants and more than 50% in the those with sufficient level of PA. In the group with insufficient levels of PA, low values of the T - score indicating osteopenia was found in both the proximal and distal measurement sites, 71 and 80% respectively [39].

Sports training for people with spinal cord injuries is often the main factor to prevent the loss of BMD that occurs with age and due to immobilization. Physical activity based on the intensive involvement of forearms led to a significantly better condition of bone tissue in this location. According to the mechanostat theory, the effect of pressure forces generated by working muscles is local in nature, which explains the beneficial effect of the upper limbs driving a wheelchair on BMD in wheelchair rugby players. Rugby training also includes resistance exercises, which are an important element of beneficial bone loading [38]. Athletes of sports characterized by high impact forces, such as rugby, have higher BMD than non-athletes [40]. Participation in regular impact exercises is generally suggested as a way to reduce the risk of osteoporosis at a later age [33]. Most studies to date have suggested that resuming sports activity at a right time after treatment and rehabilitation is useful in preventing the loss of BMD in wheelchair athletes and can also affect their quality of life [10]. In our study, men who trained rugby had significantly better forearm bone parameters than inactive men. Additionally, we analyzed whether smoking attenuates the positive effect of physical activity. Significant differences (*p* < 0.001) in the frequency of low BMD were noted between male smokers and non-smokers with SCI. More than 70% of smokers had low BMD dis, whereas in the proximal segment, low BMD was found in all smokers. The results of the multiple forward stepwise regression analysis reveal that years of active smoking were a significant negative predictor for BMD dis with a positive effect of physical activity, fat mass, and hand grip strength. The predictor of interactions of the number of cigarettes smoked per day (negative direction) and age at injury (additive direction) was significant for T-score prox.

General population studies have shown the negative impact of smoking on BMD. However, there are very few such studies involving men with SCI. In our study, 40% of rugby players were smokers, which translated into the high prevalence of low BMD. A significant relationship (F = 87,140; *p* = 0,005) was found between mean BMD and smoking in active and inactive men with SCI. Smokers had the lowest BMD values. In contrast, among rugby players with SCI, smoking attenuated the positive effect of active lifestyles on BMD.

A meta-analysis conducted by Ward et al. [19] showed that smokers had significantly reduced bone mass compared to non-smokers who had never smoked and those who had smoked in the past. BMD deficits were particularly evident in the hip, where the bone mass of smokers was one-third SD lower than in non-smokers. The adverse effects of smoking on the health of athletes are therefore wider than just the risk of lung cancer or poorer physical capacity. This topic requires more detailed research and analysis.

Studies have shown that BMD also depends on body composition [40]. In the case of athletes, the fraction of tissue components in body mass is closely related to the type of sport practiced, training routines, and training experience. In our study, most of the rugby players had a normal BMI or were overweight, which may have been caused by higher muscle mass. There were no cases of obesity among wheelchair players, which, as studies show, is a common occurrence in people with spinal cord injuries and related to insufficient physical activity [16]. In our study, there was no significant relationship of FFM with bone parameters. However, regression analysis showed a significant positive effect of fat mass in association with physical activity and hand grip strength on BMD dis. Previous studies have demonstrated the effect of body composition on BMD and BMC. Elite rugby players are characterized by body mass index (BMI) similar to that in obese people [38], but they have significantly lower body fat, higher fat-free mass, while their skeleton is more frequently exposed to stress induced by training. Studies suggest the key role of lean body mass in maintaining bone strength and resistance to fractures [40]. These results were not confirmed in our study.

Previous studies of the determinants of BMD in men have shown a significant positive effect of muscle strength on bone parameters [13]. Athletic training leads to increased muscle strength. In our study, active men with SCI had a significantly better HGS score than inactive men. Furthermore, HGS had a significant effect on BMD dis. Previous studies have also found that long-term practicing of rugby, from adolescence onwards, is associated with significant increases in BMC, BMD, and bone size in numerous skeletal locations [35, 40, 41]. Musculoskeletal adaptations represent a response to training loads. However, it is worth noting that smoking can limit the beneficial effect of sports training on bone health. Furthermore, studies have demonstrated that significant changes in body composition are observed at later stages of sports training. Increased fat mass and lower fat-free mass can have a negative effect on the power-to-weight ratio, and can therefore generate lower forces on the skeletal system [35].

This study makes an important contribution to this area of research. In men with SCI especially those physically active who exercise regularly, early detection of the risk of low BMD allows for taking effective preventive measures and reducing the risk of osteopenia, osteoporosis, and consequent fractures. It should be emphasized that our study focused on bone parameters of the non-paralyzed upper limb, which is a rare approach as most previous studies have focused on lower limbs affected by paraplegia. The major strength of the study is that a reliable and accurate research methodology was used. The research was conducted by a highly-qualified team with many years of research experience in the field. All data were collected using well-selected and internationally recommended research tools. One of the study limitations is the relatively small yet sufficient size of the study group. It cannot provide a full representation of the population of men with SCI at this age. The body tissue composition was evaluated only by the anthropometric method as it was impossible to perform bioelectrical impedance testing.

## Conclusions

Rugby can be considered a sport that has a beneficial effect on forearm BMD. Physical activity itself and in interactions with fat mass and hand grip strength (positive direction) had a significant positive effect on bone health in the non-paralyzed upper limb. Active smoking may reduce the protective role of physical activity in improving bone health. The findings of the study confirm the importance of promoting physical activity in patients with SCI. Regular physical activity improves the condition of forearm bone tissue, which is important for wheelchair locomotion. The findings of this study highlight the importance of recommending and supporting smoking cessation strategies for people living with SCI.

## Data Availability

The datasets used and/or analyzed during the current study are available from the corresponding author on reasonable request.

## Abbreviations

AIS: ASIA Impairment Scale
BMI: Body mass index
BF: the percentage of the body fat
BMD: Bone mineral density
BMC: Bone mineral content
DXA: Dual-energy X-ray absorptiometry
FM: Fat mass
FFM: Fat-free mass
dis: Distal part of forearm
HGS: Hand grip strength
ISCD: International Society for Clinical Densitometry
Prox: Proximal part of forearm
SCI: Spinal cord injuries
